# Case Report: Identification of Mutations in *LAMP2* in Two Chinese Infants With Danon Disease

**DOI:** 10.3389/fgene.2020.589838

**Published:** 2021-01-11

**Authors:** Luyan Zhang, Fan Yang, Mei Chen, Ming Zhou, Tianwei Qian, Mohammed Omer Mujtaba, Abdul Haseeb Mohammed, Jie Yin, Xueying Cheng, Jinlong Chen, Yuming Qin, Shiwei Yang

**Affiliations:** Department of Cardiology, Children’s Hospital of Nanjing Medical University, Nanjing, China

**Keywords:** Danon disease, *LAMP2*, *MYH7*, hypertrophic cardiomyopathy, genetics

## Abstract

Danon disease (DD) is a monogenic lysosomal storage disorder characterized by cardiomyopathy, skeletal myopathy, and variable degrees of intellectual disability. It is caused by a deficiency of lysosomal-associated membrane protein 2 (*LAMP2*). Two unrelated boys who presented with severe hypertrophic cardiomyopathy and elevated levels of liver enzymes, and were diagnosed with Danon disease at a very young age, were investigated. One boy was diagnosed at 4 months old and died soon after; his mother also died of hypertrophic cardiomyopathy shortly after his birth. Another developed hypertrophic cardiomyopathy at 3 months old but reported no significant cardiovascular symptoms during more than 5 years follow-up. Genetic screening found compound variants of *LAMP2* and *MYH7* in both of them. This report highlights the clinical heterogeneity in DD. The timely identification of *LAMP2* mutation plays a critical role in their treatment and family counseling.

## Introduction

Danon disease (DD) is a rare monogenic lysosomal storage disorder that was first reported in 1981 (Danon et al., 1981). It is caused by loss-of-function mutations in the *LAMP2* gene that encodes for lysosome-associated membrane protein-2, lower levels of which will lead to disrupted autophagy (Nascimbeni et al., 2017). The *LAMP2* gene maps to the chromosome region Xq24, its open reading frame consists of 1,233 nucleotides and encodes 410 amino acids. Exons 1–8 and part of exon 9 encode the luminal domain; the remainder of exon 9 encodes a transmembrane domain and a cytoplasmic tail (Nishino et al., 2000; Cenacchi et al., 2020). Most of the mutations in *LAMP2* identified in DD so far include small deletion insertions and nonsense or splicing alterations, resulting in a loss of transmembrane or cytoplasmic domains, which causes *LAMP-2* protein deficiency (Endo et al., 2015).

DD is characterized by the triad of cardiomyopathy, skeletal muscle dystrophy, and intellectual disability. Clinical manifestations are variable but generally hypertrophic cardiomyopathy determines the course and prognosis of the disease. Conduction system abnormalities are common findings at presentation such as ventricular preexcitation and Wolff-Parkinson-White (WPW). As expected in the X-linked condition, cardiac symptoms in males usually begin in infancy/childhood or adolescence with rapid progression toward heart failure while in females usually present with later onset and slower progression (Cenacchi et al., 2019).

Here we report two male infants with early-onset hypertrophic cardiomyopathy and persistent abnormal levels of serum alanine transaminase (ALT) and aspartate aminotransferase (AST). Next-generation sequencing revealed compound variants of *LAMP2* and *MYH7* in both of them. One boy reported no significant cardiovascular symptoms during more than 5 years of follow-up while another died at 4 months old, shortly after diagnosis.

## Case Presentation

### Patient 1

**FIGURE 1:**
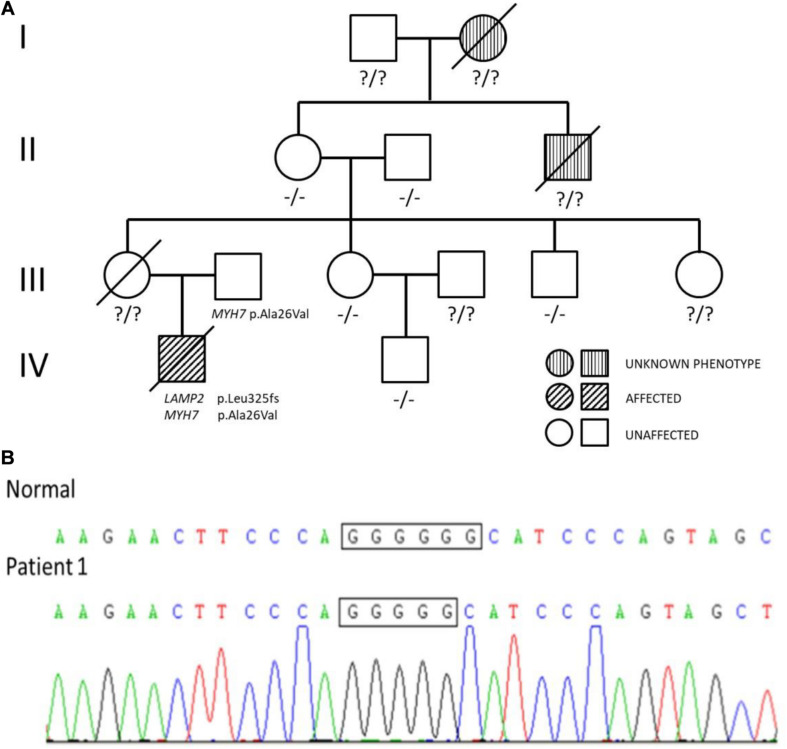
**(A)** Pedigree analysis of the family of patient 1. The arrow points out the proband. Circles correspond to female. Squares correspond to male. The mutation was indicated −/− if negative and ?/? if untested. **(B)** Genetic testing of patient 1 showed a guanine deletion at position 973 (c.973delG) in exon 8 of the *LAMP2* gene.

The first patient was a 4-month-old boy, urgently referred to our hospital for a cough and shortness of breath in January 2011. He was a premature test-tube baby and born prematurely weighing 2.45 kg. The boy had cyanosis when crying and sucking. The boy had an enlarged tongue and suffered from dysphagia. His mother had a history of hypertrophic cardiomyopathy and died shortly after giving birth to him. His mother’s uncle and grandmother both died young of unknown causes in their thirties (Figure 1A).

**FIGURE 2:**
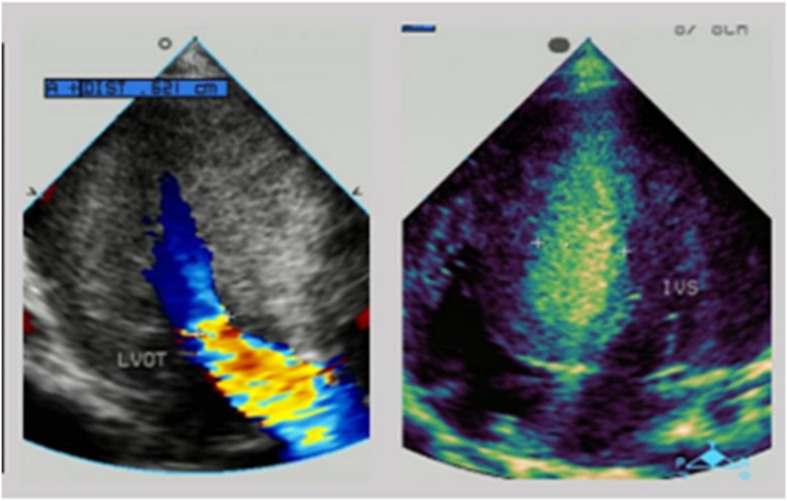
The echocardiogram of patient 1: substantial left ventricular hypertrophy with a maximal thickness in the posterior wall of the left ventricle of 10 mm and a ventricular septal thickness of 15 mm, and obstruction in the left ventricular outflow tract.

There was low cardiac sound and a II/VI grade systolic murmur at the left border of the sternum. The neurological evaluation was conducted and revealed decreased muscle strength. A routine 12-lead electrocardiogram showed a sinus rhythm and voltage criteria consistent with biventricular hypertrophy. The echocardiogram showed substantial left ventricular hypertrophy with a maximal thickness in the posterior wall of the left ventricle of 10 mm and a ventricular septal thickness of 15 mm, and obstruction in the left ventricular outflow tract (Figure 2). The laboratory studies showed elevated levels of transaminases [ALT: 154 U/L, AST: 283 U/L, and lactic dehydrogenase (LDH): 1,617 U/L]. Creatine phosphokinase (CK) levels were also elevated at 869 U/L and serum troponin-T levels were found higher at 0.083 ng/ml.

Medical treatment did not go well. The boy was transferred to ICU. Two weeks after admission, the boy had a sudden ventricular fibrillation. Although the ECG showed that the ventricular fibrillation disappeared after rescue, the child had fallen into a coma without spontaneous breathing. Auscultation revealed low heart sound with arrhythmia. His pupillary light reflex disappeared and the pupils were dilated. Neither tendon reflex nor superficial reflex existed. His father finally decided to give up treatment.

The diagnosis of hypertrophic cardiomyopathy could not fully explain the elevated levels of transaminases and decreased muscle strength in the boy. After fully-informed consent was given, next-generation sequencing was performed and found compound variants of *LAMP2* and *MYH7* in the boy. There was a guanine deletion at position 973 (c.973delG) in exon 8 of the *LAMP2* gene (Figure 1B), and so the patient was diagnosed with Danon disease. It was predicted to result in a frame shift of the amino acid sequence p.Leu325fs, resulting in a stop codon at residue p.345 and a premature termination of translation. Bioinformatic analysis based on Mutation Taster suggested that the variant was disease causing. Another heterozygous variant c.77G > A (p.Ala26Val) in exon 2 of *MYH7* was inherited from his phenotypically normal father. Other tested family members did not carry the variants without any symptoms (Figure 1A).

### Patient 2

**FIGURE 3:**
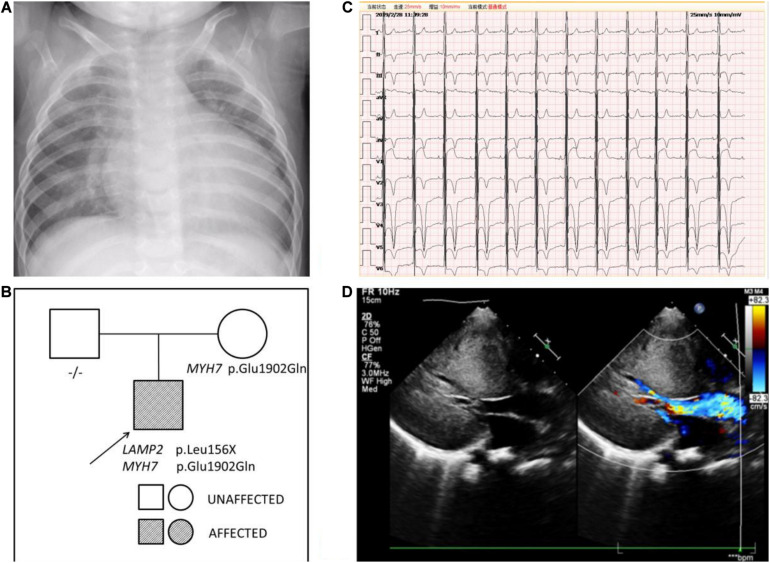
**(A)** The chest x-ray of patient 2 demonstrated an enlarged cardiac silhouette. **(B)** Pedigree analysis of the family of patient 2. The arrow points out the proband. Circles correspond to female. Squares correspond to male. The mutation was indicated -/- if negative. **(C)** The ECG of patient 2 showed biventricular high voltage. **(D)** The echocardiogram of patient 2 revealed severe hypertrophic cardiomyopathy with impaired diastolic function (E/A: 0.8) and normal systolic function (LVEF: 87.6%).

Another boy was first evaluated in hospital for a heart murmur at the age of 3 months. He was diagnosed with hypertrophic cardiomyopathy and was given metoprolol as treatment. At the age of 6 months, he came to medical attention in our hospital because of a cough and wheezing in October 2014. There was a II/VI grade systolic murmur at the left border of the sternum and wheezing in the lungs. Neurological examination showed decreased muscle strength. ECG indicated a sinus rhythm with left ventricular high voltage and ST-T changes. A chest x-ray demonstrated an enlarged cardiac silhouette (Figure 3A). The boy was referred for 2-dimensional echocardiography and 64-slice dynamic contrast-enhanced CT. Echocardiography revealed normal biventricular dimensions, the interventricular septum and posterior wall of the left ventricle was predominantly thickened with obstruction in the left ventricular outflow tract and normal systolic functions. The right ventricular size and function were within normal limits, as were the valve structure and function. CT revealed morphologic findings similar to those of the echocardiogram without thrombi and dilation of the pulmonary artery and veins. Laboratory tests showed impaired liver function and continuously abnormal liver enzyme levels, with a maximum ALT level of 228 U/L, serum AST level of 243 U/L, and LDH level of 1,411 U/L. Plasma amino acids, urine organic acids, and serum ceruloplasmin were normal.

**FIGURE 4:**
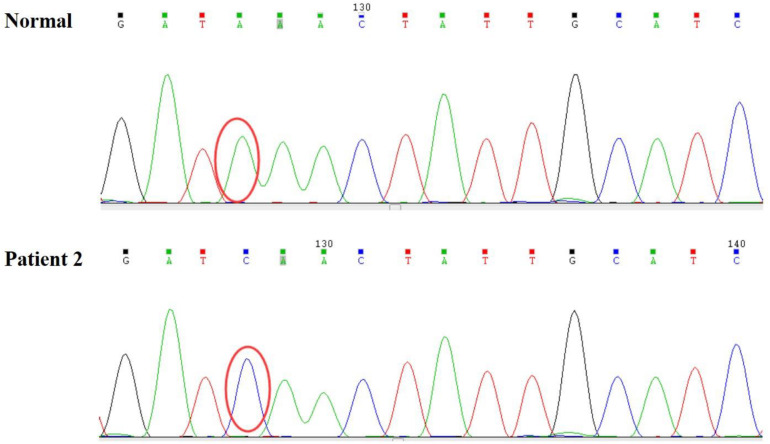
A hemizygous c.467T > G (p.Leu156X) mutation in exon 4 of *LAMP2* was identified in the patient.

Persistent abnormal levels of liver enzymes led us to suspect the presence of metabolic disease. Skeletal muscle biopsy was not performed due to refusal by the parents. The following genetic analysis identified two compound heterozygous variants of *LAMP2* and *MYH7* in this boy. A hemizygous c.467T > G (p.Leu156X) in exon 4 of *LAMP2* was identified in the patient causing a premature stop codon, thereby confirming a diagnosis of Danon disease (Figure 4). This mutation was not detected in his biological parents and *de novo*. The heterozygous c.5704G > C (p.Glu1902Gln) in exon 4 of *MYH7* found in the patient was inherited from his asymptomatic mother. No variant was detected in the phenotypically normal father (Figure 3B).

The patient was treated with metoprolol. During 5 years follow-up, he has reported no significant cardiovascular symptoms. As of our last contact with him, the boy had myopia and astigmatism with normal fundus and no intellectual disability. However, his lower limb strength had decreased and he could not run. Transaminase levels were still higher than normal. The ECG result progressed to biventricular high voltage (Figure 3C). Besides, echocardiography revealed that interventricular septum, the posterior wall of the left ventricle and the anterior wall of the left ventricle were predominantly thickened with impaired diastolic function (E/A: 0.8) and normal systolic function (LVEF: 87.6%) (Figure 3D).

## Discussion

Danon disease is a rare X-linked condition characterized by cardiomyopathy, skeletal myopathy, and variable degrees of intellectual disability. Although both hemizygous males and heterozygous females can be affected, clinical manifestations can vary widely (Cenacchi et al., 2019). Cardiac symptoms in females usually present with later onset and slower progression while males usually begin to present in infancy/childhood or adolescence with rapid progression toward heart failure (Cenacchi et al., 2019). In our report, patient 1 died at a very young age of 4 months, and patient 2 was diagnosed with DD at 3 months old.

As far as we all know, DD is a monogenic disease caused by mutations in *LAMP2* that encodes for lysosome-associated membrane protein-2, the deficiency of which will cause autophagy disruption, leading to an impaired fusion of lysosomes to autophagosomes, and the biogenesis of lysosomes (Nascimbeni et al., 2017; Cenacchi et al., 2020). Interestingly, we identified two variants in *LAMP2* and *MYH7*, respectively. In patient 1, a frame shift variant [c.973delG (p. Leu325fs)] of *LAMP2* and a *MYH7* variant [c.77G > A (p.Ala26Val)] were identified. Although c.77G > A (p.Ala26Val) of *MYH7* was found in HCM before (Arai et al., 1995), the boy’s father who carried the variant was healthy, suggesting that the *MYH7* variant seemed unlikely to cause cardiomyopathy. The mutation c.973delG (p.Leu325fs) of *LAMP2* was reported in a 10-year-old girl who was diagnosed with DD and underwent cardiac transplantation due to severe heart failure 9 months after presentation (Hedberg Oldfors et al., 2015) (Table 1). It was predicted to cause disease by Mutation Taster. Besides, the mutation was only identified in the proband boy and absent in other healthy family members. Therefore, we thought that the *LAMP2* mutation was the leading cause of the disease. Since his mother died of hypertrophic cardiomyopathy shortly after giving birth to him, it was reasonable to suspect that Danon disease contributed to her death and the *LAMP2* mutation in the patient came from her. The early-onset cardiomyopathy with rapid progression in both the reported girl and the boy in this case suggested that the mutation predicted a malignant clinical course of DD with a high rate of disease-related death at an early age and implantation of a left ventricular assist device or heart transplantation was necessary.

**TABLE 1:** Clinical features of four patients with mutations in *LAMP2* from different reports.

Mutation	c.973delG		c.467T > G	
Reference	Patient 1	Hedberg Oldfors et al., 2015	Patient 2	Yang et al., 2005
Age/sex	4 months old/male	10 years old/female	3 months old/male	13 years old/male
Clinical manifestation	Short of breath decreased muscle strength	Palpitations	Decreased his lower limb strength	Palpitations near syncopal episodes
ECG	Sinus rhythm, voltage criteria consistent with biventricular hypertrophy	Sinus rhythm, preexcitation. QRS-duration 128 ms, very large voltages, widespread ST-Twave changes	Sinus rhythm, left ventricular high voltage, ST-T changes	WPW syndrome, supraventricular tachycardia, chronic atrial fibrillation
Echo cardiography	Substantial left ventricular hypertrophy	Left atrial enlargement	Left ventricle predominantly thickened	HCM with moderate concentric hypertrophy
	Obstruction in the left ventricular outflow tract	Generalized cardiac hypertrophy	Obstruction in the left ventricular outflow tract	No left ventricular outflow tract obstruction
Laboratory examination	Levated levels of transaminases. creatine phosphokinase levels, serum troponin-T levels	Slightly elevated levels of AST and Troponin T.lnitial NT-pro-BNP level was 5,800 ng/L.	Levated levels of transaminases. nornal lasma amino acids, urine organic acids and serum ceruloplasmin	Elevated several liver enzymes, creatine kinase levels, troponin 1 levels
Outcome	Dead	Cardiac transplantation	Without cardiovascular symptoms	Without heart failure

In patient 2, c.5704G > C (p.Glu1902Gln) in exon 39 of *MYH7* was reported in HCM (Chiou et al., 2015). However, the boy’s mother was asymptomatic with normal electrocardiogram, echocardiography, physical examination, and blood biochemistry. Taking this into consideration, the *MYH7* variant was not the leading cause in this case. The variant c.467T > G (p.Leu156X) in exon 4 of the *LAMP2* gene has been reported to be associated with Danon disease (Yang et al., 2005). Yang et al. described a 13-year-old male patient who presented with palpitations and near syncopal episodes with ECG showing WPW syndrome, supraventricular tachycardia, and non-sustained ventricular tachycardia successively (Table 1). He finally accepted a biventricular implantable cardiac defibrillator. In addition, combined with the continuous elevated levels of liver enzymes and the decrease of lower limb muscle strength in the boy, we believed that *LAMP2* mutation was the cause of the disease.

Mutations of *MYH7* which encodes the β-myosin heavy chain are one of the most common causes of hypertrophic cardiomyopathy (Marian and Braunwald, 2017). As mentioned above, the two *MYH7* variants identified are not the main cause of disease. Whether they were involved in the progression of cardiomyopathy or contributed to the severity of cardiomyopathy in the two boys is unknown. More cases and functional trials are needed.

In conclusion, genetic testing should be recommended for unexplained myocardial hypertrophy with persistent abnormalities in liver function. Early diagnosis is very important to prevent sudden cardiac death and to find relatives at risk of developing the disease. Besides, c.467T > G (p.Leu156X) of the *LAMP2* may predict a poor prognosis. For patients who may die at an early age, implantation of a left ventricular assist device (LVAD) and/or heart transplantation are essential options.

## Data Availability Statement

The original contributions presented in the study are included in the article/supplementary material, further inquiries can be directed to the corresponding author/s.

## Ethics Statement

Written informed consent was obtained from the participants’ legal guardian/next of kin for the publication of any potentially identifiable images or data included in this article.

## Author Contributions

LZ, FY, and MC contributed to the case presentation, composition, and editing of the manuscript. MZ and TQ cared for the patient and collected samples. MM, JY, and AM cared for the patient and collected the patient’s information. XC, JC, YQ, and SY assisted in manuscript editing. All authors have read and approved the final manuscript.

## Conflict of Interest

The authors declare that the research was conducted in the absence of any commercial or financial relationships that could be construed as a potential conflict of interest.
